# Outbreak of pneumococcal pneumonia among shipyard workers in Marseille, France, January to February 2020

**DOI:** 10.2807/1560-7917.ES.2020.25.11.2000162

**Published:** 2020-03-19

**Authors:** Nadim Cassir, Laurence Pascal, David Ferrieux, Christiane Bruel, Christophe Guervilly, Stanislas Rebaudet, Kostas Danis, Lora Kopec, Florence Fenollar, Emmanuelle Varon, Véronique Vig, Jean-Luc Lasalle, Lauriane Ramalli, Pierre Michelet, Jean-Christophe Lagier, Nicolas Persico, Philippe Brouqui, Philippe Malfait, Philippe Parola

**Affiliations:** 1 University Hospital Institute -Méditerranée Infection (IHU), Marseille, France; 2 Aix Marseille University, IRD, AP-HM, MEPHI, Marseille, France; 3 These authors contributed equally to this work; 4 French National Public Health Agency (Santé publique France), Marseille, France; 5 Regional Health Agency of Provence-Alpes-Côte d’Azur (ARS Paca), Marseille, France; 6 Service de Médecine Intensive - Réanimation, APHM, Hôpital Nord, Marseille, France; 7 Center for Studies and Research on Health Services and Quality of Life (CEReSS), Aix-Marseille University, Marseille, France; 8 Hôpital Européen, SESSTIM, Aix-Marseille Univ, INSERM, IRD, Marseille, France; 9 French National Public Health Agency (Santé publique France), Saint Maurice, France; 10 Aix Marseille University, IRD, AP-HM, SSA, VITROME, Marseille, France; 11 National Reference Laboratory for pneumococcus, Créteil, France; 12 Service des Urgences Adultes, Hôpital de la Timone, UMR MD2, Aix-Marseille Université, Marseille, France; 13 Service des Urgences Adultes, Hôpital Nord, Marseille, France

**Keywords:** Outbreaks, invasive pneumococcal disease, pneumonia, shipyard, contractors

## Abstract

We report the third outbreak of pneumococcal pneumonia within one year among workers in European shipyards. During January and February 2020, 37 cases of pneumonia were identified in a shipyard in Marseille, south-eastern France. Outbreak control measures were implemented, including a mass vaccination campaign with 23-valent pneumococcal polysaccharide vaccine targeting all shipyard workers. Given the high mobility of shipyard workers, coordinated responses between European public health institutes are necessary to avoid further outbreaks.

On 27 January 2020, the University Hospital Institute-Méditerranée Infection (IHU) reported to the regional health agency (ARS) six cases of pneumococcal pneumonia that had occurred within the 10 previous days among contractors at the shipyard of Marseille, south-eastern France.

Here we describe the preliminary results of the ongoing investigations, the control measures implemented, and discuss further measures that should be undertaken at a larger scale.

## Epidemiological investigations

Considering that this number of pneumococcal pneumonia cases was unusually high, health authorities declared a pneumococcal pneumonia outbreak at the shipyard of Marseille. The regional office of the National Public Health Agency, IHU, ARS and the National Reference Laboratory (NRL) for Pneumococcus in Créteil, formed a multidisciplinary team to investigate and control the outbreak, in collaboration with the management and occupational health services of the shipyard and the shipowner.

The first cases were interviewed to collect initial information on demographics, risk factors for pneumococcal pneumonia, work tasks and on-site working conditions in order to describe them. The IHU team carried out retrospective case finding through a review of emergency room reports and laboratory notifications since 3 January 2020. A survey study could not be carried out because of the language barrier, limited access to ships for security reasons and interval start of workers.

## Shipyard workforce and organisation

Nearly 3,200 workers accessed on a daily basis a cruise liner undergoing renovation. Some of the workers were employed directly by the shipyard of Marseille or local contractors but most of them were employed by numerous foreign subsidiaries or subcontractors. Between 3 January and 7 February, the workforce and crews represented 5,823 people of 102 different nationalities: the largest groups were Italian (25%), Romanian (11%), Polish (10%), Filipino (8%) and Ukrainian (5%) citizens. An indoor and outdoor renovation process started on 3 January 2020 and ended on 7 February 2020. The workers were accommodated on three ships: the cruise liner undergoing renovation with 880 crew members and two other ferries moored in the dry dock area with 124 and 105 crew members. All crew members worked as hotel staff to accommodate the other workers. The three ships were crowded and the workers were exposed to fumes, dust and solvents on the renovation site. The workers were mostly involved in interior outfitting.

## Case definition

A confirmed case was a worker or a crew member of the cruise liner in the shipyard of Marseille with a clinical and radiological diagnosis of pneumonia from 3 January 2020 and *Streptococcus pneumoniae* isolated from blood or endobronchial samples or pneumococcal antigen detected in the urine. If there was no laboratory confirmation, the case was defined as probable.

## Outbreak description

Until 6 February 2020, we recorded 18 probable and 19 confirmed cases. All cases except for one were male with a median age of 39 years (range: 22–66 years). All cases were identified among workers, except for four crew members. The onset of disease ranged between 9 January and 6 February (Figure). Medical history was collected for 17 cases of pneumonia: half of them were smokers, four had a chronic respiratory disease, two had a heart disease and one was diabetic. As at 12 March, no new cases have been identified after 6 February, 35 cases have recovered and probably left Marseille and two cases were still hospitalised.

**Figure:**
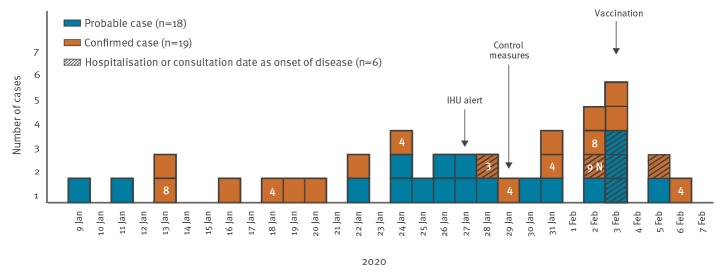
Confirmed and probable cases during a pneumococcal disease outbreak at a shipyard by disease onset date, Marseille, south-east France, January–February 2020 (n = 37)

IHU: University Hospital Institute.

Serotype number is shown in white.

Among the 37 cases of pneumonia, 18 were hospitalised, including five in an intensive care unit. Seventeen cases were co-infected with a virus, including six cases with influenza virus.

Nine cases of pneumococcal pneumonia were confirmed by positive urinary pneumococcal antigen, four by isolation from blood or endobronchial samples and six by both detection methods. All 10 *S. pneumoniae* isolates were sent to the NRL for Pneumococcus for serotyping. Serotyping was performed with the use of latex particles sensitised with pool, group, type and factor antisera provided by the Statens Serum Institut (Copenhagen, Denmark). The following serotypes were identified from blood cultures of eight patients, serotype 3 (n = 1), 4 (n = 4), 8 (n = 2) and 9N (n = 1), and an additional serotype 4 from the endobronchial sample of another patient. Whole genome sequencing of isolates and analysis of nasopharyngeal swabs are ongoing. 

## Control measures

On 29 January, the clinical management of the cases was organised in collaboration with the medical team on board of the cruise liner, the emergency medical help services, IHU and the emergency units of Marseille Public University Hospital (AP-HM) and other hospitals. Systematic antibiotic treatment with amoxicillin was prescribed for all symptomatic patients [1]. Antibiotic prophylaxis was not recommended. It was recommended that staff of the cruise liner reinforce hand hygiene, use of surgical masks for people with respiratory symptoms and medical consultation for any person with respiratory signs and/or fever.

On 3 and 4 February, a team of 20 health workers implemented a mass vaccination campaign for the 4,300 workers and crew members still on board of the three ships on 31 January. This campaign included requesting consent forms and providing flyers in six different languages (English, Italian, Polish, Romanian, Russian and Spanish). At that time, the serotypes of the strains isolated from blood culture were unknown. As we had to act fast before the ship left Marseille, we decided to use the 23-valent pneumococcal polysaccharide vaccine (PPV23) to protect against a larger number of pneumococcus serotypes. For logistical reasons, co-vaccination for seasonal influenza could not be implemented. In parallel, the medical team of the cruise ship administered vaccinations on board between 2 and 6 February and continued to do so after the departure of the ship to Italy. By 25 February, 1,460 persons had been vaccinated.

The French health authorities issued a communication through the European Union Early Warning and Response System (EWRS) on 7 February to inform and facilitate potential case finding in Italy and across Europe. To date no other cases have been notified by European countries*.*

## Background

*Streptococcus pneumoniae* is a Gram-positive bacterium with nearly 100 serotypes currently recognised [2]. Through the colonisation of the nasopharyngeal tract, the bacteria can cause mild infections such as otitis media, pneumonia and invasive pneumococcal disease (bacteraemia or meningitis) [2]. Infections caused by *S. pneumoniae*, including invasive pneumococcal diseases (IPD), remain a significant public health concern worldwide. In 2018, the incidence of IPD in the general population in France was 7.9 cases per 100,000 inhabitants [3]. The onset of pneumococcal infections may be related to the combination of high carriage rates, the genetic adaptability of pneumococci and their ability to shift from a commensal to a pathogenic interaction with its host [4].

## Discussion 

Regarding community-acquired pneumonia in adults, outbreaks of pneumococcal disease have been described in close and crowded environments (prisons, care facilities, shelters) [5]. Several risk factors related to these outbreaks have been identified, including smoking, people 65 years and older, poor nutritional status, immunosuppression, several comorbidities and respiratory viral infections [6]. IPD is a well-known complication of influenza [7].

Pneumococcal outbreaks had not been reported in shipyards before 2015 [5]. In 2015, nine cases of serious pneumococcal disease were identified in Northern Ireland [8]. Three IPD cases were reported at a shipyard in Singapore in 2017 [9]. In 2019, one outbreak occurred in Norway and a second in Finland with 20 and 37 cases, respectively [10,11]. Notably, in the latter, three different *S. pneumoniae* serotypes were isolated from blood cultures of 25 cases. Over the course of a year, Europe has recorded three outbreaks in different shipyards, raising concerns over the risk of pneumococcal disease in people working in crowded and polluted environments. Welders are recognised to be at greater risk to develop IPD owing to their exposure to metal fumes. Vaccination with PPV23 is recommended in the United Kingdom for this population [12-14]. France does not have specific guidelines for this occupational group. 

The cases identified in the shipyard of Marseille presented several risk factors for pneumococcal disease including exposures to respiratory irritants, smoking and viral coinfections. Workers lived and worked in crowded environments. Several occupations that facilitated exposure to respiratory irritants were identified among the pneumonia cases: technicians (n = 7), interior outfitters and installers (n = 6), fire-guards (n = 2), managers (n = 2), painter, a welder and a carpenter. Seven of them reported direct exposure to dust, solvent or metal fumes. As in the 2019 outbreak in Finland [11], several different serotypes were present. Serotype distribution differs across countries depending on vaccination policies, among other factors. For example, serotype 4 has not been prevalent in France since the implementation of children vaccination with PCV13 [15]. The question remains whether the onset of pneumococcal pneumonia in these outbreak may be attributable to a propagated outbreak with spread from person to person and/or a shift from commensal to pathogen under favourable conditions (workplace exposure, viral infections).

Outbreak control measures were difficult to implement as workers came from different countries with different vaccination strategies, spoke different languages and were employed by several contactors. In addition, the goal of vaccinating 4,300 workers and crew members was difficult to achieve as the vaccination campaign had to be completed within 5 days because of the departure date of the ship and of the workers.

## Conclusion

Given the high mobility of shipyard workers across several ports in Europe, coordinated responses between public health institutes are warranted to avoid future outbreaks. Health and occupational authorities need to reinforce occupational hygiene measures and to consider recommending both pneumococcal and seasonal influenza vaccination for shipyard workers who are exposed to respiratory irritants.
